# Supercritical Fluid Extraction Kinetics of Cherry Seed Oil: Kinetics Modeling and ANN Optimization

**DOI:** 10.3390/foods10071513

**Published:** 2021-06-30

**Authors:** Ivana Dimić, Lato Pezo, Dušan Rakić, Nemanja Teslić, Zoran Zeković, Branimir Pavlić

**Affiliations:** 1 Faculty of Technology, University of Novi Sad, Blvd. Cara Lazara 1, 21000 Novi Sad, Serbia; ivana.dimic@live.com (I.D.); dusan.rakic@uns.ac.rs (D.R.); zzekovic@tf.uns.ac.rs (Z.Z.); 2 Institute of General and Physical Chemistry, University of Belgrade, Studentski Trg 12-16, 11000 Belgrade, Serbia; latopezo@yahoo.co.uk; 3 Institute of Food Technology, University of Novi Sad, Blvd. Cara Lazara 1, 21000 Novi Sad, Serbia; nemanja.teslic@fins.uns.ac.rs

**Keywords:** cherry seed oil, supercritical fluid extraction, kinetics modeling, mass-transfer model, artificial neural network

## Abstract

This study was primarily focused on the supercritical fluid extraction (SFE) of cherry seed oil and the optimization of the process using sequential extraction kinetics modeling and artificial neural networks (ANN). The SFE study was organized according to Box-Behnken design of experiment, with additional runs. Pressure, temperature and flow rate were chosen as independent variables. Five well known empirical kinetic models and three mass-transfer kinetics models based on the Sovová’s solution of SFE equations were successfully applied for kinetics modeling. The developed mass-transfer models exhibited better fit of experimental data, according to the calculated statistical tests (*R*^2^, SSE and AARD). The initial slope of the SFE curve was evaluated as an output variable in the ANN optimization. The obtained results suggested that it is advisable to lead SFE process at an increased pressure and CO_2_ flow rate with lower temperature and particle size values to reach a maximal initial slope.

## 1. Introduction

The food industry is known for generating large amounts of food waste, which stands for an easily available and cheap resource of high-value compounds beneficial for human health [1]. Fruit by-products have been studied in recent years with a focus on industrial utilization and optimization of extraction parameters for yield enhancement of bioactive components and obtainment of high-value extracts, which can later be used in food products [2]. Seeds and kernels, as a common stream of fruit-processing by-products, are often used as an unique source of oils rich in polyunsaturated fatty acids, tocopherols, carotenoids, phytosterols and squalene which can be applied in numerous industry fields [3,4].

In recent years, the focus has been on developing modern extraction techniques primarily emphasized on shorter processing time, reduction of hazardous organic solvents, better extraction effectiveness, while simultaneously being cost-effective and eco-friendly and these techniques are named green extraction techniques [5,6]. The tendencies in research are moving towards the replacement of conventional extraction techniques by novel approaches in order to decrease environmental pollution related to organic solvents by using green solvents, such as water, glycerol, vegetable oils, supercritical fluids and ionic liquids [7] and natural deep eutectic solvents.

Supercritical fluid extraction (SFE) has been recently introduced in food, pharmaceutical, cosmetic, nutraceutical, chemical, environmental and fuel industries [3,6]. A supercritical state of a fluid indicates that its temperature and pressure are exceeding their critical points. As a consequence, its physical and thermodynamic characteristics change, and it is often said that supercritical fluids have properties between liquid and gas. They have liquid-like density, which produces solvating power as similar to liquids and gas-like viscosity and diffusivity, which leads to higher mass transfer [8,9,10]. The most frequently used fluid is CO_2_, since it is risk-free, non-toxic, readily available in high purity, eco-friendly, low-cost solvent with moderate critical parameters (31.4 °C and 74.8 bar) [6,10]. SFE is advantageous for the extraction of particular compounds that are soluble in supercritical CO_2_, due to its polarity that is very similar to toluene. In this way, the extraction of lipophilic active compounds is greatly eased [1,11].

The outcome of the SFE process is influenced by different process parameters such as pressure, temperature, solvent flow rate, but also particle size, porosity, nature of the plant material and moisture [12]. Primary condition for a successful supercritical fluid extraction lies in a solubility of a desired active compound in supercritical CO_2_. Different parameters, such as pressure and temperature, can make an impact on the yield and composition of the extracts, since CO_2_ solvation power depends considerably on heating and pressurization. Having information about these effects can be utilized for optimization and economical assessment of the process, which is further used to transfer process to industrial level and to design and optimize an industrial plant [12,13]. Due to its better transfer abilities, the diffusion into solid matrix is enhanced [7]. Additionally, CO_2_ is simply removed at ambient conditions and one of the advantages of this solvent is the possibility of re-using, assisting in cost savings and makes the process practical at industrial scale [1].

SFE is based on internal (pressure and temperature) and external (sample matrix and ambient conditions) characteristics of the supercritical fluid, which leads to the selection of the correct experimental design [14]. Process sustainability is influenced by thermodynamic properties, whereas the size and scale-up of equipment depend on transport properties and chemical kinetics parameters, so the process design and evaluation can be performed. By using process design, it is possible to reduce energy loss and material costs [15].

Considering the outcome of the SFE process, optimization can be done in favor of achieving the utmost total extract yield, the superior concentration of desired compounds and/or particular components in the extracts or their fractions and the optimum recovery of the compounds from the raw material [6]. Kinetic models, built on heat and mass transfer correlation or mass balance relations, can be applied for resolving mathematical analysis of the SFE process and scaling-up from laboratory to pilot scale [16]. Kinetic curves represent functions of extracted mass depending on time, flow rate or solvent-to-feed-mass ratio and are later used for the process scale-up and estimation of the production expenses. Extraction curves consist of three parts according to different mass transfer mechanisms. Firstly, it can be divided into constant extraction rate (CER) period which depends on convective mass transfer and it is controlled by thermodynamic equilibrium of the solute. The next period is falling extraction rate (FER) period, determined by its slower rate since the diffusion mechanism collides with convection. The last period is the diffusion-controlled period (DC), which occurs after the recovery of all the extractable solutes. The principal mechanism is based on the diffusion of residual solutes from solid matrix to extraction medium [13].

The transport phenomena in the SFE process can be explained through mathematical modeling of such processes. Known mathematical models used to explain the SFE kinetics could be categorized in several categories: empirical models, models based on heat-transfer analogy, mass-transfer based models, or a combination of those models [17]. The SFE modeling approach finds its use in various industrial applications since it could be utilized for scale-up and/or to improve process parameters. Recently, artificial neural network (ANN) approach has been used extensively for simulation and optimization of SFE processes in order to determine the best set of process parameters, which would provide the highest yield [18,19,20]. This approach has limitation for optimization of SFE processes on industrial scale since total extraction yield is determined at the end of the process, i.e., until the complete exhaustion of the plant material. It is not feasible from an economical point of view to perform SFE until the complete exhaustion of plant matrix. Pavlic et al. [16] proposed the alternative approach for optimization of SFE processes which aim to maximize initial slope of kinetic curves, which is associated with solubility-controlled extraction phase and industrial scale processes. However, all these works were based on ANNs mimicking the natural neural system using computer software and they relied on several advantages, such as nonlinearity, adaptively, generalization, model independence, easy to use and high accuracy. The cherry seed oils extraction using different extraction processes [21] or supercritical CO_2_ extraction of tea seed oil [22] and cherry seed oils extraction [23] were already mentioned in the literature. However, these studies were limited in several SFE experiments which does not provide thorough information about the influence of SFE parameters on extraction kinetics and yield.

The fundamental objective of this investigation was the development of various empirical and mass-transfer based models for fitting the cherry seed oil SFE process. Furthermore, the determination of the SFE parameters’ (such as pressure, temperature, CO_2_ flow rate and particle size) effect on kinetic curves and flexible model parameters were investigated. The Box-Behnken experimental design with 15 regular and 6 additional runs was employed with an intention to offer a thorough set of information on how SFE parameters affect extraction kinetics, while the influence of the process parameters was assessed by one-factor-at-a-time (OFAT) approach. The final task of this research was the initial slope evaluation of the extraction curves through the SFE artificial neural network optimization with a view on maximizing the initial mass transfer rate of the extraction curves.

## 2. Materials and Methods

### 2.1. Plant Material and Chemicals

The industrial by-products of cherry seeds were received from the domestic cold-pressed oil factory, PAN-UNION d.o.o. (Novi Sad, Serbia). The plant material was immediately milled in a hammer mill (ABC Engineering, Pančevo, Serbia) and subjected to the SFE experiments. Mean particle size of the milled sample was determined by sieving through the vibro-sieve set (CISA Cedaceria Industrial, Barcelona, Spain) and the calculated mean particle size of the milled plant material used in experiments was 741 μm. The same vibro-sieve set was used to divide raw material to <0.8 and >0.8 mm particle size fractions, which were later used to examine particle size influence on SFE process.

Carbon dioxide (99.9%) used in SFE experiments was acquired from Messer Technogas A.D., Novi Sad, Serbia.

### 2.2. Supercritical Fluid Extraction (SFE)

The supercritical fluid extraction experiments (SFE) of cherry seed oil were performed through high pressure extraction at laboratory scale (HPEP, NOVA-Swiss, Effretikon, Switzerland) described by Pavlic et al. [24]. The high pressure extraction apparatus consists of a gas cylinder with CO_2_; diaphragm type compressor with a pressure range up to 100 MPa; an extractor with a heating jacket as a heating medium (internal volume 200 mL, maximum operating pressure of 70 Mpa); a separator with a heating jacket as a heating medium (internal volume 200 mL, maximum operating pressure of 25 MPa); pressure control valve; temperature regulation system; gas flow regulation valves. In individual experiments, the extractor vessel was loaded with 130.0 ± 0.01 g of cherry seeds. Consecutively, after 15, 30, 45, 60, 90, 120, 180 and 240 min of the process, the total extraction yield (Y) was measured for every sample and this data was used to determine the dynamics and kinetics of cherry seed oil extraction. In the first experimental part, Box-Behnken experimental runs (15 experiments), which has been successfully used in optimization of SFE experiments [25], were set, in which three independent variables were varied at three levels and had three central point replicates. Pressure influence was examined at 200, 275 and 350 bar, temperature values were 40, 55 and 70 °C and CO_2_ flow rate influence was investigated at 0.2, 0.3 and 0.4 kg/h. Other SFE variables, such as mean particle size (0.741 mm) and extraction time (4 h) were kept at their constant values. The six additional runs were later added to the experimental design to examine the influence of different combinations of independent process parameters (CO_2_ flow rate, temperature, pressure and particle size). According to these results, the experimental design would give further information on extraction dynamics and how the SFE parameters influence yield by applying one-factor-at-a-time (OFAT) approach. A complete experimental design is presented in Table 1. Cherry seed oil was separated from CO_2_ at 15 bar and 25 °C in separator.

### 2.3. Mathematical Modeling of Kinetic Curves

#### 2.3.1. Empirical Models

The experimentally obtained results (15 + 6 runs in total, with the different SFE parameters) were fitted to five kinetics and three mass-transfer based models. Five frequently used empirical models which were implemented for the SFE kinetics of the cherry seed oil (CSO) are presented in Table 2. These empirical models are well-known and widely used for SFE modeling and are further described by other authors [26]. Nomenclature of the abbreviations used in all applied models is given in Supplementary Material.

#### 2.3.2. Mass-Transfer Models

The mass-transfer SFE models investigated in this study were based on the equation system proposed by Sovová [33,34]. The solution of the Sovová’s model was presented as the first mass-transfer model (designated as Model VI), which was solved by the Excel procedures presented in the paper by Cabeza et al. [34]. A few assumptions were made in order to solve the Sovová’s model: (1) a non-stationary mass balance was considered in each phase, (2) the bed porosity remains constant during the SFE process, (3) there is no diffusion transport within the extraction column, (4) the diffusion effects in the axial direction are minor and (5) the solubilization of the CSO according to Henry’s equation [34].

The simplified set of balance equations for the supercritical fluid (SCF) and the solid phase may be expressed as [34]:

(1) $$\frac{\partial C_{SCF}}{\partial t}=\frac{1}{\varepsilon }\cdot \left[-\frac{u}{L}\cdot \frac{\partial C_{SCF}}{\partial z}+K\cdot \alpha \cdot \left(C_{SCF}^{*}-C_{SCF}\right)\right]\;$$

(2) $$\frac{\partial C_{S}}{\partial t}=\frac{1}{1-\varepsilon }\cdot \left[-K\cdot \alpha \cdot \left(C_{SCF}^{*}-C_{SCF}\right)\right]\;$$

where $C_{SCF}^{*}$ is the equilibrium concentration of the extracted compound in the SCF computed by a Henry’s relation with the concentration in the solid (*C_S_*): $C_{SCF}^{*}=H\cdot C_{S}$.

An additional equation should be presented, for a global coefficient calculation, to determine the equilibrium concentration and the concentration in the SCF phase, which would include all three steps of the extraction process. This coefficient could be calculated as a function of the time where the alternation among these steps occurs [34]:

(3) $$K\cdot a=\frac{k_{SCF}\cdot a_{SCF}\cdot \frac{F}{1+\exp\left(-\left(t-t_{c_{1}}\right)\right)}}{1+\exp\left(t-t_{c_{2}}\right)}+\frac{k_{S}\cdot a_{S}}{1+\exp\left(t-t_{c_{2}}\right)}\;$$

*F* represents a correction factor (between 0 and 1). The term *k_SCF_*·*a_SCF_*·*F* presents the overall mass transfer coefficient in which internal and external diffusion are co-dominant. $t_{c_{1}}$ is the extraction time interval driven by internal diffusion and external mass transport, while $t_{c_{2}}$ is linked to external transport dictated extraction.

According to the original Sovová’s SFE model [33,35], a solution of the theoretical model was presented in the paper by Rizza [36]. The implementation of this solution was applied in Matlab code. Based on the aforementioned study, two mass-transfer models were developed and presented within this study. The second mass-transfer model of SFE process (Model VII) is a simplified model presented by only two equations that explain the extraction yield (*e*) in the two extraction periods:

(4) $$e=q\cdot y_{S},\;0\leq q\leq q_{c}\;$$

(5) $$e=x_{u}\cdot \left[1-C_{1}\cdot exp\left(-C_{2}\cdot q\right)\right],q>q_{c}\;$$

where *x_u_* is the solute weight fraction in the unprocessed solid, *q_c_* is the relative amount of elapsed solvent at the end of the first extraction period (CER) and *C*_1_ and *C*_2_ are the adjustable parameters.

The passed solvent at the end of the CER period (*q_c_*) was also calculated considering that the initial value of the second equation is equal to the first one (with q_c_ as unknown value). While parameters *C*_1_, *C*_2_, and *q_c_* were calculated, the grinding efficiency and the internal mass transfer coefficient should be evaluated according to the following equations [36]:

(6) $$r=1-C_{1}\cdot exp\left(-\frac{C\cdot q_{c}}{2}\right),\;k_{s}a_{s}=\left(1-r\right)\cdot \left(1-\varepsilon \right)\cdot \dot{Q}\cdot \frac{C_{2}}{N_{m}}$$

The parameters presented in the Equation (6) were used as the initial values of *r* and *k_s_a_s_* during the calculation of the complete model.

An improved mass-transfer model (in comparison with the Model VII), also based on the Sovová’s SFE model, was presented in the following text (Model VIII). The limiting points between the first (CER) and the second period (FER), and between the second and the third period (diffusion-controlled rate—DCR period) is calculated using the equations:

(7) $$q_{m}=\frac{r\cdot x_{u}\cdot \theta_{e}}{y_{s}}\;$$

(8) $$q_{n}=q_{m}+\gamma \cdot \theta_{i}\cdot ln\left[1-r+r\cdot exp\left(\frac{1}{\beta }\right)\right]\;$$

where *q_m_* and *q_n_* are the relative amount of elapsed solvent at the end of the CER and the FER period, respectively.

These equations are derived, with the assumption of the plug flow, without a solute-to-matrix interaction. Additionally, the solute was considered homogeneously distributed within the solid matrix, while the solvent density and the bed characteristics (such as void fraction and specific surface area) were considered invariant to the displacement of the solute from particles to solvent. The complete model equations could be written as [37]:

(9) $$e=q\cdot y_{s}\cdot \left[1-exp\left(\frac{1}{\theta_{e}}\right)\right],\;0\leq q\leq q_{m}\;$$

(10) $$e=q\cdot y_{u}-r\cdot x_{i}\cdot \theta_{e}\cdot exp\left(\frac{\beta }{\theta_{e}}\cdot ln\left(1+\frac{1}{r}\cdot \left(exp\left(\frac{q-q_{m}}{\gamma \cdot \theta_{i}}\right)-1\right)\right)-\frac{1}{\theta_{e}}\right),\;q_{m}\leq q\leq q_{n}\;$$

(11) $$e=x_{u}\cdot \left(1-\beta \cdot ln\left(\left(1-r\right)\cdot \left(exp\left(\frac{1}{\beta }\right)-1\right)\cdot exp\left(\frac{q-q_{m}}{\gamma \cdot \theta_{i}}\right)\right)\right),\;q_{m}\leq q\leq q_{n}\;$$

### 2.4. Artificial Neural Network (ANN) Optimization

Artificial neural network model is a mathematical tool widely applied for solving nonlinear problems and problems involving numerical constraints [38,39]. This research made an attempt to predict the initial slope of the SFE curve, according to the process parameters such as pressure, temperature, CO_2_ flow rate and particle size. A multi-layer perceptron model (MLP), containing three layers, was utilized for the optimization of the initial slope of the SFE process [40,41]. Experimental data from runs 1–19 (Table 1) were used in ANN simulation. All the data from the experimental work for ANN modeling was randomly divided—60% was used for training, 20% was used for testing and 20% was used for validation. For solving unrestricted ANN modeling optimization problems, Broyden–Fletcher–Goldfarb–Shanno (BFGS) algorithm was applied as an iterative method [42].

The learning cycle of ANN was based on the training data, which was additionally used to evaluate the optimal number of neurons in the hidden layer and each neuron’s weight coefficient. When learning and cross-validation curves approach zero, training can be considered successful [43].

### 2.5. Global Sensitivity Analysis

Evaluation of the relative impact of the parameters on the initial slope of the SFE curve was evaluated by Yoon’s interpretation method [44]. The Yoon’s equation was used according to the calculated ANN weight coefficients.

### 2.6. Statistical Analysis

The extraction kinetics modeling for Models I-V was conducted using MS Excel 2007, Model VI was calculated in Excel, using Cabeza’s Excel routines [34], Models VII and VIII were calculated using Matlab code estimations [36], while the ANN calculation was performed in Statistica 12.0 (StatSoft, Palo Alto, CA, USA). The investigated correlation among experimentally acquired SFE yields and the appropriately calculated values found by developed models was evaluated in terms of the sum of squared errors (SSE), coefficient of determination (*R*^2^) and average absolute relative deviation (AARD).

## 3. Results and Discussion

### 3.1. Influence of SFE Parameters

In this research, cherry seed (CS) was investigated as a plant resource for oil extraction. Primarily, moisture content and particle size were determined. The SFE parameters are widely known to influence mass-transfer from plant material in a remarkable manner and can explain SFE process kinetics. The properties of plant matrix, such as morphology, herbal part, water content, shape and porosity can be useful to model the SFE process, but the most influential parameters stated in the literature are pressure, temperature, solvent flow rate and target compound solubility [45].

The Box-Behnken design was arranged with 15 regular and 6 additional experiments to provide a detailed dataset on how the SFE parameters make an impact on the extraction kinetics. One-factor-at-a-time (OFAT) procedure was applied to evaluate the influence of the SFE parameters. Impact of pressure (200, 275 and 350 bar), temperature (40, 55 and 70 °C), CO_2_ flow rate (0.2, 0.3 and 0.4 kg/h) and particle size <741, 741 and >800 μm) on total extraction yield (Y) and kinetic parameters was also studied by OFAT approach.

**Figure 1:**
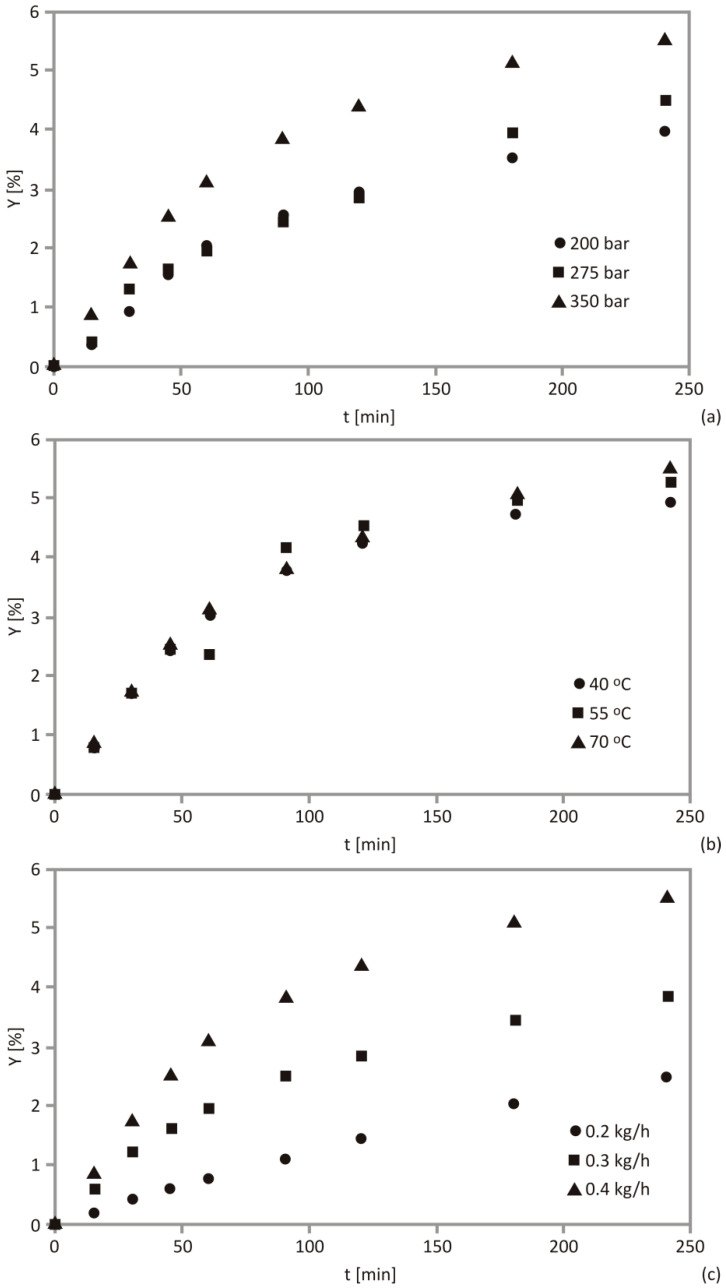
Impact of (**a**) pressure on supercritical fluid extraction (SFE) kinetics at 70 °C and 0.4 kg/h (particle size: 741 μm), (**b**) temperature on SFE kinetics at 350 bar and 0.4 kg/h (particle size: 741 μm) and (**c**) impact of CO_2_ flow rate on SFE kinetics at 350 bar and 70 °C (particle size: 741 μm).

The estimation of the pressure impact during the process was done using OFAT method, while other SFE parameters were kept constant (temperature was 70 °C; CO_2_ flow rate was 0.4 kg/h, and particle size was 741 μm), which is shown in Figure 1a. Based on the acquired results, the extract yield increased with the increase of pressure.

The increase of the applied pressure up to 350 bar showed a significant impact on Y, in comparison to pressure levels of 200 and 275 bar (Figure 1a). The pressure increase to 350 bar exhibited a positive impact to the kinetics curves, since solvation power of supercritical CO_2_ increases with pressure elevation. High pressure causes disruption of plant cells and tissues and increases extraction efficiency [46]. Similar occurrence was observed in various studies. Pavlić et al. [22] have shown that an augmentin pressure from 100 to 300 bar enhanced extraction yield of sage herbal dust. However, when the pressure was enhanced from 100 to 200 bar, the yield increase was more prominent, while further increase to 300 bar did not significantly enhance yield. An identical phenomenon appeared in wheat germ oil extraction, performed in 250–350 bar pressure range, since higher pressure values did not alter extraction yields considerably. It can be concluded that each plant material has to be studied individually with a view of industrial application, since pressure may be a prevailing factor which influences extraction kinetics [26]. Other authors reported that pressure was also the prevailing factor, individually and in interaction with other parameters, in the SFE of elderberry, raspberry, blackberry and black currant [47,48,49,50].

**Figure 2:**
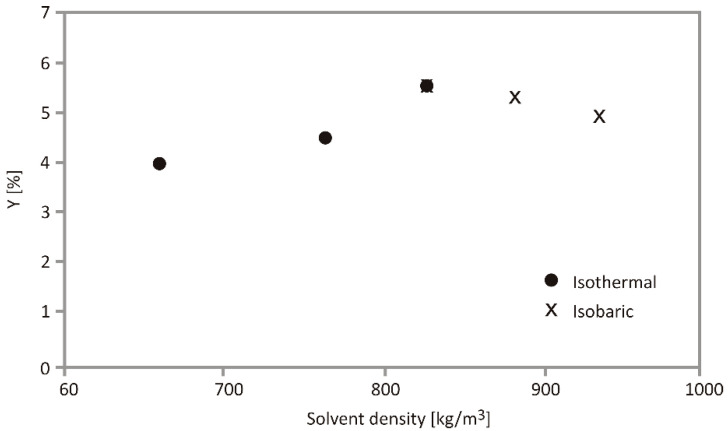
Effect of CO_2_ density on total extraction yield at isothermal (70 °C) and isobaric (350 bar) conditions.

The effect of CO_2_ density on total extraction yield at isothermal and isobaric conditions, calculated using OFAT approach is presented in Figure 2.

The increase of pressure directly influences the density of supercritical CO_2_ in terms of contribution to the solvent density increase and its ability for better dissolving the target compounds. However, the density is somewhat limited at higher pressure levels [51]. Temperature increase diminishes CO_2_ density and solubility, but simultaneously, at the higher temperatures, solutes become more volatile and the final results could not always be easily predicted. This phenomenon was investigated in similar temperature conditions for coriander seed extraction and increase from 40 to 70 °C has decreased CO_2_ density [52]. The global yield of açaí berry oil depended predominantly on carbon dioxide density, which was influenced by a combination of pressure and temperature [53]. The economic benefits of the SFE process at an elevated pressure level should be further investigated in every case study to more precisely define the extract yield and energy consumption equilibrium [54].

The temperature effect at a constant set of SFE parameters (350 bar, 0.4 kg CO_2_/h and 741 μm) can be observed in Figure 1b. It could be inferred that the temperature showed a limited impact since the obtained Y values reached 5.31; 5.54 and 4.93% at 40, 55 and 70 °C, respectively. The SFE curve trend was similar for all the observed temperatures, with an insignificant increase of the extraction rate, when elevating the temperature level from 55 to 70 °C. Obtained results are in agreement with a study focused on SFE of raspberry seed oil on temperatures between 30 and 60 °C, showing minor impact of this parameter on oil yield [48]. Additionally, this variable did not express crucial influence on wheat germ oil yield in range from 40 to 60 °C and it can be concluded that SFE may be performed on lower temperatures to reduce energy waste [26].

The increase of temperature (with a fixed pressure level) contributed to the decrease of the supercritical CO_2_ density, and thus its solvation power. In contrast to pressure, temperature exerts influence on both solvent and solute properties. When the temperature is increased, the solute vapor pressure is increased simultaneously, which could lead to better extraction rate [55], consistent with our conclusions. This synergistic effect was proven in the SFE extraction of blackberry and cranberry pomace [49,56]. Additionally, this phenomenon relies on the nature of the solute and plant material and may not be suitable for crude extracts [13]. For example, higher yields were recovered at 80 °C for cloudberry and black currant seeds, but yield for bilberry seeds was higher at 50 °C, because complex intramolecular interactions in compound mixtures make the extraction yield more difficult to estimate [57].

The thermodynamic parameters (such as density and solvent solubility) and transport properties (viscosity and internal diffusivity) are controlled by pressure and temperature. On contrary, convective mass-transfer phenomena, axial dispersion and accumulation in the supercritical phase depend on flow rate and solvent velocity [51]. Concentration gradient is predicted to be higher with increasing solvent flow rate, thus the extraction rate would be improved, which could be observed in Figure 1c. These conclusions correspond to previous studies [54]. Similar flow rate increase from 0.2 to 0.4 kg/h enhanced the yield of sage herbal dust, wheat germ and grape seed extracts [22,26,58]. It should be emphasized that higher operational costs may be caused by increased flow rate, which should be estimated for each case from an industrial point of view [45]. It is also important to observe that high flow rates cause scarce contact time between solutes and solvent and may decrease the yield [16]. The CER period would define economic reasons for the supercritical CO_2_ consumption within the industry.

**Figure 3:**
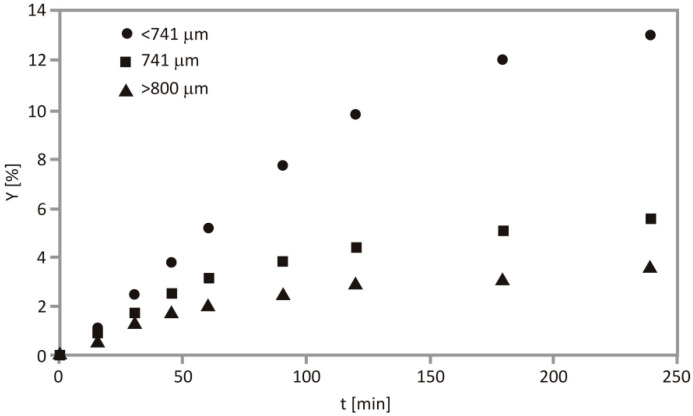
Effect of particle size on SFE kinetics at 350 bar, 70 °C and 0.4 kg/h.

The impact of particle size on the SFE process was assessed at constant pressure (350 bar), temperature (70 °C) and CO_2_ flow rate (0.4 kg/h) and is depicted in Figure 3. According to the results, downsizing the particle size from >800 to <800 μm caused an increase of Y. Smaller particles were characterized by greater surface area per unit of volume and possess a larger amount of free oil which is easily accessible for dissolution; consequently, the internal mass-transfer resistance is lowered. Additionally, the core of the cherry seeds, which is rich in oil, may be concentrated in fraction containing smaller particles [35]. The disadvantage of particle size reduction is aggregation and channeling of the particles, which leads to lower extraction rate caused by reduced fluidized bed velocity and filter congestion [16]. This trend was reported by Pavlić et al. [22], in the range <200 μm, which had negative impact on raspberry oil yield, while fraction between 200 and 400 μm has been proven for obtaining the highest yield.

### 3.2. SFE Kinetics Modeling

It has been assumed that there are no other studies conducting an investigation on the SFE kinetics of CSO. This study aims to contribute to this research field by evaluating the SFE kinetics using the following process parameters: pressure (200, 275 and 350 bar), temperature (40, 55 and 70 °C) and CO_2_ flow rate (0.2, 0.3 and 0.4 kg/h) during extraction (0, 15, 30, 45, 60, 120, 180 and 240 min). Five common empirical equations and three mass-transfer models were applied to fit acquired experimental results. The adequacy of fit among experimental data and suggested models was examined using statistical parameters, such as sum of squared errors (SSE), coefficient of determination (*R*^2^) and average absolute relative deviation (AARD). For all implemented models, remarkably high values of *R*^2^ and low SSE and AARD (Tables S1 and S2) suggested the adequate fit. The best fit was achieved by Model VI as it had the lowest SSE and AARD values and the highest *R*^2^ (mean for all experiments: 0.052; 0.043 and 0.998, respectively). The recent SFE research on wheat germ oil has shown similar results in comparable experimental conditions [26].

Flexible parameters obtained from Model I, *Y_∞_* and *k* were affected by the SFE parameters (pressure, temperature, CO_2_ flow rate and particle size) observed in this study (Table 3). The parameter *Y_∞_* ranged from 2.97 to 6.48% while *k* ranged between 0.002 and 0.014 min^−1^. The largest *Y_∞_* value was gained at 200 bar, 55 °C and 0.4 kg/h CO_2_ (run 7), while the highest value of *k* was achieved at 350 bar, 40 °C and 0.4 kg/h CO_2_ (run 17). Three SFE parameters that were modified in experimental design caused a complex influence on customizable model parameters, thus it was important to precisely express how the particular SFE parameter affects the process and it was done according to OFAT approach (Table 3). The pressure exhibited a positive influence on *k*. Furthermore, *Y_∞_* also increased with pressure increase, since solvent density and solvation power were intensified at higher pressures. Temperature also showed positive impact on *Y_∞_* for Model I (Table 1 and Table 3). This may indicate how the increase of the temperature, while keeping other SFE parameters constant, showed notable influence towards the vapor pressure of the solute, rather than expressing negative influence on solvent density.

Model II was obtained through the modification of Model I by adding the correction term *b*. A similar trend of the SFE parameters impact on *Y_∞_* was obtained through Model II, because pressure, temperature and CO_2_ exerted positive influence on *Y_∞_* (Table 3). *Y_∞_* value was between 2.98 and 6.51%, *a* was in the range of −0.015–−0.002, while parameter *b* was in the range from −0.014 to 0.026 min^−1^.

Additionally, it can be noted that SFE parameters expressed a similar effect on *Y_∞_* and this tendency can be recognized in all other models (Table 3). Pressure and CO_2_ flow rate contributed to the declining trend of the adjustable parameter *a*, while temperature had a rather irrelevant effect. In the case of *b*, pressure and temperature showed a negative impact (Table 3).

Model III parameters *Y_∞_* and *k* were influenced by pressure, temperature and solvent flow rate almost likewise. The parameter *Y_∞_* was calculated from 3.97 to 9.00% while *k* was between 79.72 and 525.40 min^−1^. All SFE parameters positively affected *Y_∞_* according to the OFAT approach.

**Figure 4:**
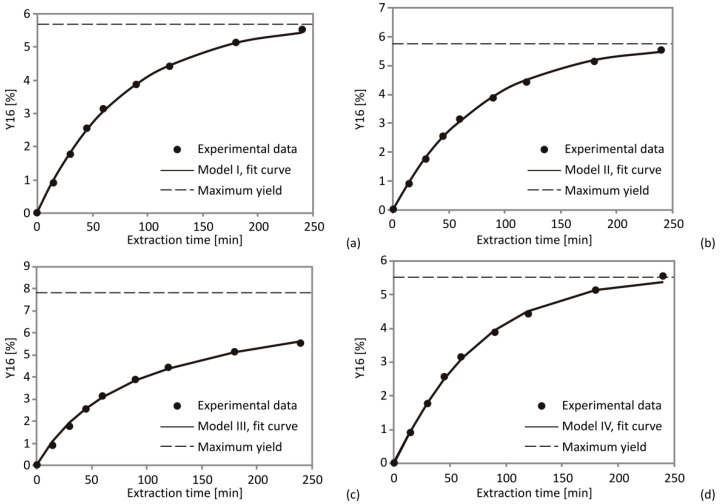
Extraction curves with experimental and model data obtained for Models I–IV at the following SFE conditions: 350 bar, 70 °C and 0.4 kg CO_2_/h, comparison of the experimental data with (**a**) Model I, (**b**) Model II, (**c**) Model III and (**d**) Model IV.

According to Figure 4d, it could be concluded that the extraction curve obtained by Model IV consists of two individual curves, which refer to extracted fractions during solubility-controlled and diffusion-controlled periods, respectively. The overall extraction curve for Model IV was derived as the sum of two separate curves for *f*_1_ and *f*_2_ extraction parts, and it could be related to the extraction curves obtained using other kinetics models (Figure 4).

Calculated parameters taken from the Model V suggested that the CER period ranged from 291.10 to 787.47 min (Table 4). The results indicated that the lowest *t*_1_ was achieved at the lowest pressure, while experiment 11, conducted on central level parameter values (275 bar, 55 °C and 0.3 kg CO_2_/h), contributed to the shortest CER period (Table 4), while the temperature had a negative impact on *t*_1_ (Table 4), which was consistent with the former research [26]. A notable amount of oil in the sample clarifies the relatively long *t*_1_ in comparison with the extraction time. The second extraction step (falling extraction rate; FER period) was described by parameter *t_i_* with values between 2.64 and 5.91 min (Table 4). It could be observed that the FER period did not finish after 240 min for a number of runs, suggesting that the total extraction time applied in experiments was not enough to entirely release the extract from the plant material. However, the SFE process on the industrial level does not depend entirely on the FER period and would be terminated as soon as CER period is finished [59].

The OFAT analysis showed that temperature revealed a negative influence on *Y_∞_*, while solvent flow rate had a positive effect on adjustable parameter *Y_∞_* (Table 4). The Model V results suggested that *Km* values were between 70.92 and 233.92, aligning with previous SFE process studies with different plant materials in similar experimental conditions [26,52]. Asymptotic yield (*Y_∞_*) is used in empirical models as an adjustable variable providing significantly high values of calculated *Y_∞_*.

These models are easily calculated since they have fewer parameters, but they do not describe the process adequately, according to *R*^2^, AARD and SSE. On the other hand, mass transfer models use more parameters and have an advantage over empirical models.

**Figure 5:**
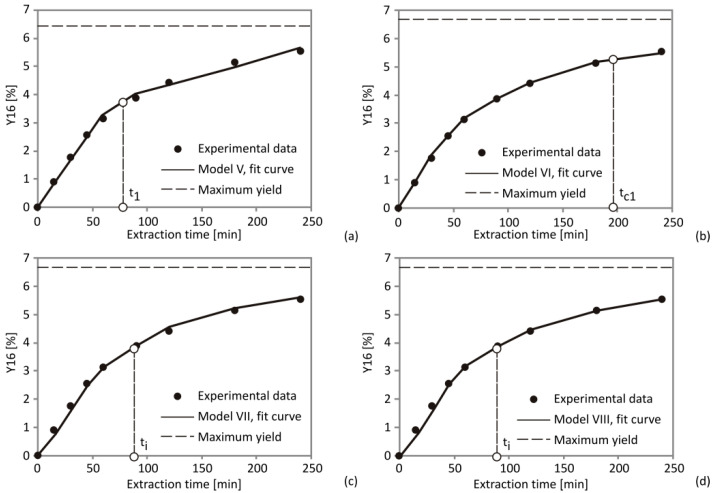
Extraction curves with experimental and model data obtained for Models V–VII at the following SFE conditions: 350 bar, 70 °C and 0.4 kg CO_2_/h, comparison of the experimental data with (**a**) Model V, (**b**) Model VI, (**c**) Model VII and (**d**) Model VIII.

The mass-transfer based model proposed by Sovová [35] and solved by Cabeza et al. [34], (Model VI) was the most compatible fit to the experimental data (Figure 5 and Table S2). One of the advantages of this model is the ability to accurately identify the process periods: CER, FER and DCR. The CER period was depicted by parameter *t_c_*_1_, which predominantly depends on the SFE parameters (Table 4). The increase of pressure and the augment of solvent flow rate estimated by the OFAT method caused an increase of *t_c_*_1_ and also a reduction of *t_c_*_2_ (Table 4). This can be attributed to the fact that mass-transfer resistance becomes lower with a higher solvation power and concentration gradient. This trend was dependent on the positive impact of pressure on the diffusion constant *k_SCF_a_SCF_* (min^−1^) (CER) (due to the modification of solvent density and solutes vapor pressure), and a negative impact on diffusion constant *k_s_a_s_*, (min^−1^) (FER), which was noticed in the case of cupuassu butter extraction process as well [59]. On the contrary, temperature elevation contributed to the extension of CER period. This model also showed the best fit in the SFE of raspberry seeds [24].

The mass-transfer based model suggested by Sovová [35] exhibited an adequate fit to the experimental data (Table 5). The CER period was defined by *t’* parameter, which was affected by SFE parameters in a great manner (Table 5). Lowering of *t_i_* was caused by pressure and solvent flow rate increase, determined by OFAT method (Table 5).

### 3.3. ANN Optimization

The optimization of SFE process parameters leading towards oil recovery is usually performed with an idea to increase the value of *Y*. However, recent research mentions that scale-up from experimental results to industrial level would benefit from the initial slope maximization [26]. In the usual approach, it could be performed by response surface methodology [26] or by an ANN calculation [22]. An important parameter which describes solubility-controlled phase (CER) is the initial slope of the SFE curve. Optimization of this parameter can later be used in the industrial process. The experimental results were used to obtain the initial slope, which was later used as an input variable for the ANN modeling.

**Figure 6:**
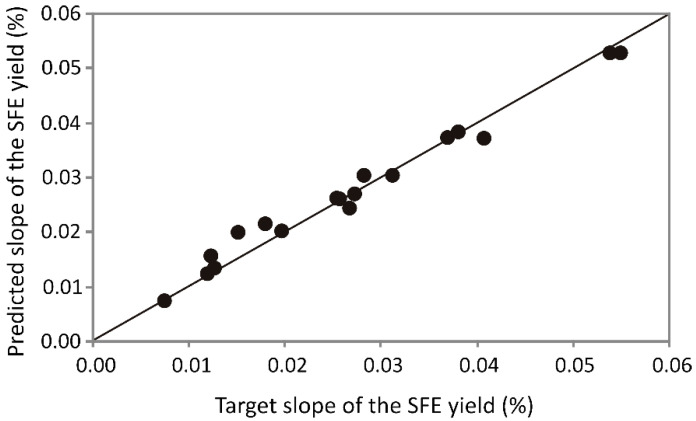
Experimentally measured and ANN model predicted values of the initial slope determined from kinetic curves.

Depending on high values of *R*^2^ (0.995 during the training period) and low values of SOS (Table S3), the optimal number of hidden neurons was 6 (network MLP 3-6-1) and it was used to calculate the slope of FSE curve. The ANN model was proven to successfully predict experimental values of the slope during supercritical extraction in CO_2_ for the majority of experimental runs, as presented in Figure 6. The ANN model was complex (with 31 weights-biases) according to the high nonlinearity of the system. The *R*^2^ values between experimental measurements of the initial slope of the SFE curve and the ANN model outputs during the training cycle of the ANN calculation was 0.995. The elements of *W*_1_ and *B*_1_, *W*_2_ and *B*_2_ matrices, used for evaluation of the ANN model are presented in Tables S4 and S5, respectively. The quality of the model fit was verified and the residual analysis of the created model was proved by an especially high *R*^2^ (Table S6).

The ANN optimization of the CSO during the SFE process was performed using the initial slope calculated from the experimental data. The initial phase of SFE process is controlled by solubility phase and can be presented through initial slope obtained from ANN model calculation. CER period is defined as linear part of the SFE curve, which is described by the initial slope. Total extraction yield is often determined after long extraction time, hence the optimization of the SFE process could be bypassed by ANN approach, according to this factor. Additionally, the extraction period of presented experiments was set to 240 min, but a number of runs exceeded this period.

The ANN model was employed to fit experimental data and obtain the initial slope of the extraction curves. Model adequacy can be confirmed by the coefficient of determination (*r*^2^). Remarkably high *r*^2^ (0.995) and low error term indicate the suitable fit between initial slope obtained experimentally and the ANN model. The elevated goodness-of-fit tests SSE (2.54·10^−4^) and AARD (0.049) proved the accuracy of the ANN model.

### 3.4. Global Sensitivity Analysis—Yoon’s Interpretation Method

**Figure 7:**
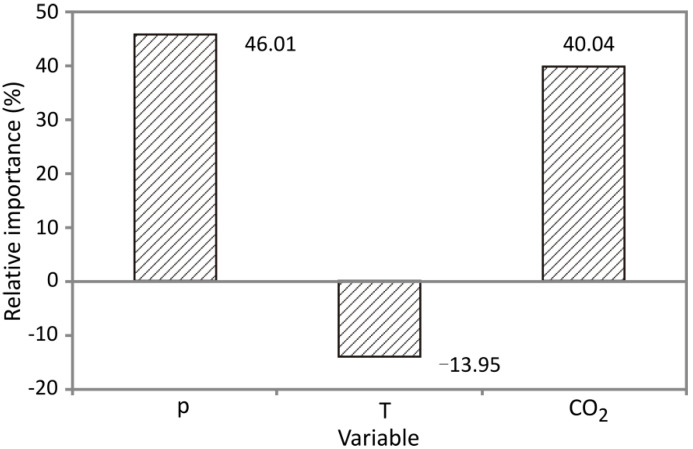
The relative importance of the FSE parameters on the *Y* value, estimated using Yoon’s interpretation formula.

This paragraph focuses on studying the impact of pressure, temperature and solvent flow input variables, identified by the Yoon’s interpretation method and ANN. According to Figure 7, pressure was the most prestigious parameter with relative importance of +46.01% making it the most dominant parameter, while supercritical CO_2_ flow rate impact reached a relative influence of +40.04%. The influence of temperature was negative, slightly lesser in absolute value in comparison to other parameters, reaching a level of −13.95%. Increased pressure causes shorter distance between molecules and the interaction between cherry seed oil and CO_2_ becomes intensified. As a consequence, oil becomes more soluble in CO_2_ and the extraction is improved in CER period [24]. Importance of flow rate is significant due to the fact that the initial extraction phase relies upon solubility and continuous flow of the fresh solvent, which causes quicker dissolution of the solute because of the high concentration gradient. Rise of temperature decreases CO_2_ density which would overcome the effect of increased solute’s vapor pressure and decrease extraction rate [52]. Similarly, pressure and flow rate exhibited a positive influence on initial slope in SFE of coriander seeds, while temperature had a negative influence [52]. It may be concluded that influence of the extraction parameters depends on the active compounds being recovered, since Pavlić et al. [22] have shown that all three aforementioned variables expressed positive influence in SFE of sage herbal dust.

Optimal conditions determined using ANN for the obtaining highest value of initial slope were pressure of 350 bar, temperature of 40 °C and flow rate of 0.4 kg/h. According to the ANN model, the suggested temperature is 40 °C, which agrees with the observed results, due to the negative effect that temperature exhibited on initial slope. Similarly, optimal values for raspberry seed and coriander seed oil extraction showed that SFE should be conducted at higher pressure and flow rate, but lower temperature, which can be advantageous from an economic point of view because it reduces required heating energy [24,52].

## 4. Conclusions

Cherry seeds stand for an underused by-product of fruit processing, thus, their valorization in order to recover bioactive compounds and latter application in food, cosmetics and pharmaceutical products may have economic significance in industry. In this work, influence of independent SFE parameters (pressure, temperature, flow rate and particle size) was evaluated after a series of performed experiments using expanded Box-Behnken design. The SFE conditions of 350 bar, 50 °C and 0.4 kg CO_2_ were used to obtain the highest extract yield. In order to fit kinetic curves, five frequently used empirical models and three mass-transfer models were applied. Among the selected models, the mass-transfer model (Model VI) proved to accordingly fit the experimental results and it was shown that the most influential parameters were pressure and flow rate, which had a positive effect on *Y*, while temperature had a rather negative impact. In addition, ANN were applied to calculate initial mass transfer rate, since it exemplifies the initial phase of the extraction process. In future perspective, cherry seed SFE extracts could be compared with extracts recovered by conventional and other modern extraction techniques regarding yield and chemical profile responsible for the bioactive value of this food by-product.

## Figures and Tables

**Table 1:** Box-Behnken experimental design with 15 + 6 runs, with three independent SFE parameters: pressure, temperature and CO_2_ flow rate. The additional six experiments (runs 16–21) were performed to evaluate the effects of these variables and also the particle size of the cherry seed oil (CSO).

Run	Factor 1		Factor 2		Factor 3		Density	Particle Size
	Pressure		Temperature		CO_2_ Flow Rate			
	(bar)		(°C)		(kg/h)		(g/cm^3^)	(µm)
Box-Behnken Experimental Design								
1	−1	200	1	70	0	0.3	658.95	741
2	1	350	0	55	−1	0.2	881.30	741
3	0	275	−1	40	1	0.4	894.80	741
4	0	275	1	70	−1	0.2	762.35	741
5	0	275	−1	40	−1	0.2	894.80	741
6	1	350	1	70	0	0.3	826.30	741
7	−1	200	0	55	1	0.4	754.10	741
8	−1	200	0	55	−1	0.2	754.10	741
9	1	350	−1	40	0	0.3	934.90	741
10	0	275	1	70	1	0.4	762.35	741
11	0	275	0	55	0	0.3	830.45	741
12	−1	200	−1	40	0	0.3	839.90	741
13	0	275	0	55	0	0.3	830.45	741
14	0	275	0	55	0	0.3	830.45	741
15	1	350	0	55	1	0.4	881.30	741
Additional Experiments								
16	1	350	1	70	1	0.4	826.30	741
17	1	350	−1	40	1	0.4	934.90	741
18	−1	200	1	70	1	0.4	658.95	741
19	1	350	1	70	1	0.4	826.30	741
20	1	350	1	70	1	0.4	826.30	<800
21	1	350	1	70	1	0.4	826.30	>800

Experiments 1–19 were used in extraction kinetics modeling and ANN simulation.

**Table 2:** Frequently applied empirical models implemented for fitting of cherry seed oil SFE.

No	Model Equation	Reference
Model I	$Y=Y_{\infty }\cdot \left(1-e^{-k\cdot t}\right)$	[27]
Model II	$Y=Y_{\infty }\cdot \left(1-e^{\left(a\cdot t+b\right)}\right)$	[28,29]
Model III	$Y=Y_{\infty }\cdot \frac{t}{k+t}$	[30,31]
Model IV	$Y=Y_{\infty }\cdot \left[1-\left(f_{1}\cdot e^{-k_{1}\cdot t}+f_{2}\cdot e^{-k_{2}\cdot t}\right)\right]$	[32]
Model V	$Y=Y_{\infty }\cdot G\cdot \frac{t}{t_{1}}$, for $t\leq t_{1}=\frac{G}{K_{m}\cdot \dot{q}}$	[33]
	$Y=Y_{\infty }\cdot \left[1-\left(1-G\right)\cdot e^{\frac{t-t_{1}}{t_{i}}}\right]$, for $t\geq t_{1}$	

**Table 3:** Calculated adjustable parameters of Models I–IV used for SFE modeling.

	Model I		Model II			Model III		Model IV				
Run	*Y*_∞_	*k*	*Y*_∞_	*a*	*b*	*Y*_∞_	*k*	*Y*_∞_	*f*_1_	*k*_1_	*f*_2_	*k*_2_
	(%)	(min^−1^)	(%)			(%)	(min^−1^)	(%)		(min^−1^)		(min^−1^)
Box-Behnken Experimental Design												
1	5.13	0.002	4.97	−0.002	0.001	5.61	503.21	5.29	0.997	0.002	0.010	0.280
2	4.32	0.009	4.28	−0.010	0.010	6.19	139.24	4.31	0.996	0.009	0.015	0.036
3	2.97	0.013	2.99	−0.012	−0.012	3.97	88.93	3.42	0.641	0.006	0.361	0.025
4	3.86	0.004	3.68	−0.005	0.008	6.43	392.84	3.69	0.708	0.005	0.300	0.004
5	3.02	0.007	2.98	−0.008	0.010	4.57	192.46	4.14	0.969	0.006	0.034	−0.006
6	4.07	0.011	4.11	−0.011	−0.010	5.62	111.32	4.12	0.980	0.011	0.010	0.003
7	6.48	0.003	6.51	−0.003	0.000	8.99	492.99	7.08	0.994	0.002	0.010	0.099
8	4.63	0.002	4.63	−0.002	0.007	4.63	525.40	4.63	0.997	0.002	0.010	0.002
9	4.88	0.012	4.80	−0.012	0.026	6.85	115.48	4.70	0.916	0.013	0.131	0.011
10	5.42	0.007	5.58	−0.007	−0.014	8.05	194.20	10.61	0.901	0.002	0.105	0.033
11	4.61	0.007	4.55	−0.007	0.007	7.04	211.43	10.55	0.696	0.001	0.309	0.009
12	4.78	0.003	4.37	−0.004	0.011	7.75	477.20	7.75	0.022	−0.006	0.983	0.002
13	3.95	0.008	3.94	−0.008	0.002	5.87	178.90	3.99	0.023	0.001	0.980	0.008
14	3.81	0.007	3.80	−0.007	0.001	5.71	189.60	3.92	0.116	0.003	0.885	0.008
15	5.71	0.012	5.66	−0.012	0.016	7.84	101.30	5.66	1.006	0.012	0.010	0.012
Additional Experiments												
16	5.71	0.013	5.76	−0.013	0.005	7.81	94.30	9.10	0.469	0.001	0.537	0.015
17	5.13	0.014	5.10	−0.015	0.019	6.81	79.72	9.13	0.364	−0.001	0.644	0.013
18	4.42	0.009	4.32	−0.010	0.024	6.39	142.30	9.18	0.582	0.000	0.431	0.011
19	5.15	0.003	5.18	−0.003	−0.001	7.49	489.91	7.48	0.607	0.001	0.392	0.004

**Table 4:** Evaluated adjustable parameters of Models V and VI used for SFE modeling.

	Model V					Model VI			
Run	*Y*_∞_	*G*	*K_m_*	*t*_1_	*t_i_*	*k*_f_	*k*_s_	*t_c_*_1_	*t_c_*_2_
	(%)	(min^−1^)	(%)	(min)	(min)	(min^−1^)	(min^−1^)	(min)	(min)
Box-Behnken Experimental Design									
1	3.88	0.493	228.16	329.67	3.88	0.050	0.026	194.46	204.81
2	3.79	0.900	130.01	500.00	3.79	0.030	0.029	127.88	217.03
3	3.31	0.800	136.72	500.01	3.31	0.044	0.020	180.00	205.76
4	2.64	0.800	144.81	421.97	2.64	0.016	0.025	182.89	295.91
5	2.82	0.800	147.48	388.93	2.81	0.025	0.010	118.63	321.91
6	3.91	0.800	136.44	365.38	3.91	0.019	0.024	107.56	348.93
7	3.88	0.777	215.38	349.89	3.88	0.026	0.004	192.19	209.36
8	3.88	0.409	233.92	339.62	3.88	0.028	0.019	179.36	373.64
9	4.75	0.800	81.34	477.01	4.75	0.023	0.008	200.00	493.26
10	5.59	0.800	188.83	329.53	5.59	0.023	0.016	184.45	489.20
11	4.17	0.800	152.21	291.10	4.17	0.017	0.011	197.54	464.52
12	3.88	0.710	203.19	322.60	3.88	0.019	0.013	130.21	282.01
13	3.89	0.800	155.88	306.12	3.89	0.021	0.022	161.25	334.66
14	3.88	0.785	155.59	306.08	3.88	0.040	0.026	164.86	249.71
15	5.91	0.800	94.72	787.47	5.91	0.047	0.030	92.51	499.689
Additional Experiments									
16	5.80	0.800	70.92	446.78	5.80	0.041	0.010	195.64	334.39
17	5.71	0.782	79.28	617.99	5.71	0.040	0.028	188.37	477.63
18	3.94	0.800	76.88	361.10	3.94	0.022	0.026	128.44	417.75
19	3.88	0.632	226.28	346.98	3.88	0.028	0.019	44.56	411.69

***G***—parameter related to particle size and fragmentation; ***K_m_***—mass related coefficient; ***Y_∞_***—total yield in infinite time of extraction process (%); ***t*_1_**—time constant extraction rate (min); ***t_i_***—time of internal mass transfer (min); ***k_f_***—solvent-phase mass transfer coefficient (min-1); ***k_s_***—solid-phase mass transfer coefficient (min^−1^).

**Table 5:** Calculated adjustable parameters of Models VII and VIII applied for SFE modeling.

Sample		*c_u_*	*n*	*N_mg_*		*x_u_*		*γ*		*t_i_*	*G*	*t’*		*τ_e_*		*k_f_a*_0_		*r*	*k_s_a_s_*		*q_m_*		*τ_i_*	*β_m_*		*q_n_*
		-	-	g		kg/kg		-		min	-	min		-		-		-	-							
1		0.8	4	23.764		4		0.327		372.99	0.102	43.637		0.023		833.043		0.07	0.033		0.326		576.228	0.94		23.907
2		0.84	4	19.011		5.25		0.437		192.158	0.32	52.612		0.019		625.744		0.271	0.051		0.277		233.86	1.922		17.652
3		0.75	3	29.705		3		0.444		86.507	0.337	30		0.024		625.744		0.298	0.114		0.226		133.366	1.893		11.376
4		0.8	6	23.764		4		0.378		260.264	0.303	86.675		0.027		418.445		0.21	0.044		0.65		250.59	0.814		39.538
5		0.75	5	29.705		3		0.444		128.779	0.362	58.852		0.024		314.795		0.285	0.084		0.338		90.354	0.813		21.36
6		0.82	4	21.388		4.556		0.41		136.522	0.362	41.524		0.021		833.043		0.309	0.071		0.388		239.789	1.609		23.604
7		0.85	5	17.823		5.667		0.374		310.699	0.162	58.866		0.024		1247.642		0.117	0.043		1.049		703.984	0.705		83.137
8		0.8	6	23.764		4		0.374		472.761	0.157	90		0.018		625.744		0.081	0.031		0.325		366.712	0.616		39.529
9		0.821	4	21.269		4.587		0.464		68.689	0.41	45.964		0.024		625.744		0.294	0.151		0.381		100.475	0.874		23.14
10		0.87	4	15.447		6.692		0.378		233.799	0.242	44.185		0.027		1247.642		0.197	0.042		0.844		817.438	1.98		38.48
11		0.84	5	19.011		5.25		0.412		204.36	0.307	60		0.023		833.043		0.25	0.052		0.771		366.737	1.126		46.95
12		0.81	6	22.576		4.263		0.417		258.224	0.295	90.179		0.025		625.744		0.195	0.046		0.941		344.703	0.759		62.399
13		0.805	4	23.17		4.128		0.412		147.646	0.291	45		0.025		625.744		0.229	0.069		0.395		226.263	1.357		21.177
14		0.8	5	23.764		4		0.412		155.992	0.347	57.696		0.024		625.744		0.279	0.07		0.665		220.058	0.932		39.634
15		0.84	4	19.011		5.25		0.437		53.969	0.471	45		0.019		1247.642		0.252	0.201		0.34		119.523	0.747		28.358
16		0.855	4	17.229		5.897		0.410		89.561	0.433	43.441		0.023		1247.642		0.363	0.108		0.7		261.033	1.26		39.709
17		0.835	4	19.605		5.061		0.464		64.359	0.482	45		0.026		833.043		0.383	0.151		0.669		146.096	1.024		33.934
18		0.875	6	14.852		7		0.327		373.873	0.367	83.298		0.025		1662.241		0.315	0.025		2.342		1636.46	1.782		116.163
19		0.8	4	23.764		4		0.410		254.62	0.146	41.478		0.025		418.445		0.099	0.047		0.212		216.523	1.018		13.812
Raw Material and Extractor Properties																										
**Porosity** **-**	**m** **-**				**m_in_** **g**		**Moisture** **%**		**N_g_** **g**		**D** **m**		**L** **m**		**d_a_** **kg/m^3^**		**d_r_** **kg/m^3^**			**d_p_** **m**		**a_o_** **m^−1^**			**ρ_CO2 std._** **kg/m^3^**	
0.5	9				130		8.6		118.82		0.037		0.12		1007.555		2015.11			0.001		4048.814			1.98	

***c_u_***—asymptotic extraction yield at infinite time; ***n***—period corresponding to the end of the first extraction period; ***N_mg_***—mass of insoluble solid; ***x_u_***—concentration of oil in the untreated solid (oil/insoluble solid); ***γ***—CO_2_ to solid ratio in the bed; ***t_i_***—characteristic time of the second extraction period; ***G***—initial fraction of solute in open cells; ***t’***—time at the end of the first extraction period; ***τ_e_***—external material transport resistance; ***k_f_a*_0_**—product *k_f_ · a*_0_; ***r****—*grinding efficiency; ***k_s_a_s_***—product *k_s_ · a_s_*; ***q_m_***—*q* at the end of the first extraction period; ***τ_i_***—internal material transport resistance; ***β_m_***—coefficient; ***q_n_***—*q* at the end of the second extraction period; **m**—number of experimental points; **m_in_**—cherry seed mass; **N_g_**—total dried mass (oil + insoluble solid); **D**—reactor diameter; **L**—reactor length; **d_a_**—cherry seed apparent density; **d_r_**—cherry seed real density; **d_p_**—particle diameter; **a_o_**—specific area per unit volume of extraction bed; **ρ_CO2 std_**—CO_2_ density at standard conditions.

## Data Availability

Data is contained within this article and Supplementary Materials.

## Supplementary Materials

The following are available online at https://www.mdpi.com/article/10.3390/foods10071513/s1, Table S1. Statistical parameters used for determination of fitting quality (SSE, AARD and *R*^2^) between experimental results and applied models (I–V), Table S2. Statistical parameters used for determination of fitting quality (SSE, AARD and *R*^2^) between experimental results and applied models (VI–VIII), Table S3. ANN model summary (performance and errors), for training, testing and validation cycles, Table S4. Elements of matrix *W*_1_ and vector *B*_1_ (presented in the bias column), Table S5. Elements of matrix *W*_2_ and vector *B*_2_ (presented in the bias column), Table S6. The “goodness of fit” tests for the developed ANN model, Nomenclature.
