# Supplementary material for: Supercritical Fluid Extraction Kinetics of Cherry Seed Oil: Kinetics Modeling and ANN Optimization
Source: Foods. 2021 Jun 30;10(7):1513. doi: 10.3390/foods10071513 (PMC8307763; doi:10.3390/foods10071513)
Supplement: Supplementary file 1 [file foods-10-01513-s001.zip › foods-1240891-supplementary.pdf]

Table S1. Statistical parameters used for determination of fitting quality (SSE, AARD and R^2) between experimental results and applied models (I – V)

Run	Model I			Model II			Model III			Model IV			Model V		
	SSE	AARD	R^2	SSE	AARD	R^2	SSE	AARD	R^2	SSE	AARD	R^2	SSE	AARD	R^2
1	0.003	0.015	0.990	0.003	0.015	0.999	0.015	0.033	0.997	0.005	0.020	0.998	0.004	0.015	0.999
2	0.055	0.065	0.961	0.053	0.069	0.996	0.058	0.060	0.996	0.052	0.068	0.997	0.140	0.098	0.990
3	0.014	0.034	0.982	0.012	0.033	0.998	0.002	0.008	1.000	0.001	0.009	1.000	0.080	0.081	0.989
4	0.005	0.020	0.991	0.004	0.018	0.999	0.006	0.020	0.999	0.004	0.018	0.999	0.067	0.050	0.989
5	0.004	0.020	0.993	0.003	0.017	0.999	0.012	0.033	0.998	0.001	0.008	1.000	0.030	0.042	0.996
6	0.037	0.053	0.972	0.035	0.056	0.997	0.012	0.029	0.999	0.035	0.056	0.998	0.178	0.109	0.990
7	0.003	0.015	0.997	0.003	0.015	1.000	0.012	0.030	0.999	0.004	0.021	0.999	0.024	0.038	0.997
8	0.005	0.017	0.985	0.003	0.014	0.999	0.024	0.042	0.994	0.003	0.014	0.999	0.003	0.012	0.999
9	0.058	0.067	0.972	0.056	0.065	0.997	0.177	0.119	0.992	0.069	0.063	0.998	0.532	0.182	0.977
10	0.206	0.137	0.889	0.201	0.129	0.989	0.172	0.113	0.990	0.120	0.096	0.997	0.198	0.127	0.990
11	0.012	0.028	0.991	0.011	0.029	0.999	0.015	0.029	0.999	0.010	0.025	0.999	0.107	0.081	0.993
12	0.009	0.027	0.987	0.006	0.022	0.999	0.011	0.031	0.999	0.004	0.017	0.999	0.021	0.029	0.998
13	0.009	0.025	0.991	0.008	0.026	0.999	0.009	0.022	0.999	0.008	0.026	0.999	0.070	0.076	0.993
14	0.001	0.010	0.999	0.001	0.010	1.000	0.003	0.016	1.000	0.001	0.009	1.000	0.064	0.066	0.993
15	0.545	0.172	0.820	0.543	0.176	0.981	0.715	0.209	0.976	0.543	0.176	0.983	0.342	0.151	0.989
16	0.036	0.054	0.989	0.030	0.054	0.999	0.064	0.072	0.998	0.023	0.043	0.999	0.252	0.125	0.993
17	0.048	0.057	0.982	0.037	0.051	0.999	0.187	0.118	0.993	0.029	0.049	0.999	0.094	0.073	0.996
18	0.094	0.082	0.943	0.080	0.084	0.995	0.120	0.091	0.993	0.079	0.081	0.996	0.171	0.105	0.992
19	0.001	0.008	0.998	0.001	0.008	1.000	0.005	0.020	0.999	0.001	0.009	1.000	0.005	0.018	0.999

Table S2. Statistical parameters used for determination of fitting quality (SSE, AARD and R^2) between experimental results and applied models (VI – VIII)

Run	Model VI			Model VII			Model VIII		
	SSE	AARD	R^2	SSE	AARD	R^2	SSE	AARD	R^2

1	0.004	0.018	0.999	0.005	0.021	0.999	0.004	0.018	0.999
2	0.039	0.049	0.998	0.093	0.089	0.996	0.039	0.049	0.998
3	0.210	0.129	0.986	0.015	0.032	0.999	0.210	0.129	0.986
4	0.004	0.016	0.999	0.022	0.043	0.998	0.004	0.016	0.999
5	0.007	0.022	0.999	0.030	0.050	0.997	0.007	0.022	0.999
6	0.133	0.112	0.994	0.090	0.085	0.996	0.133	0.112	0.994
7	0.006	0.023	0.999	0.023	0.044	0.998	0.006	0.023	0.999
8	0.001	0.007	1.000	0.003	0.016	0.999	0.001	0.007	1.000
9	0.188	0.102	0.994	0.036	0.054	0.999	0.188	0.102	0.994
10	0.070	0.067	0.997	0.411	0.167	0.985	0.070	0.067	0.997
11	0.049	0.061	0.997	0.067	0.075	0.997	0.049	0.061	0.997
12	0.002	0.012	1.000	0.016	0.036	0.999	0.002	0.012	1.000
13	0.028	0.046	0.998	0.032	0.050	0.998	0.028	0.046	0.998
14	0.110	0.077	0.993	0.054	0.068	0.997	0.110	0.077	0.993
15	0.122	0.092	0.996	0.376	0.140	0.988	0.122	0.092	0.996
16	0.023	0.040	0.999	0.170	0.120	0.997	0.023	0.040	0.999
17	0.055	0.062	0.999	0.115	0.096	0.997	0.055	0.062	0.999
18	0.033	0.045	0.998	0.247	0.137	0.989	0.033	0.045	0.998
19	0.004	0.018	0.999	0.008	0.026	0.999	0.004	0.018	0.999

Table S3. ANN model summary (performance and errors), for training, testing and validation cycles

Net. name	Performance			Error			Train. algor.	Error funct.	Hidden activat.	Output activat.
	Train.	Test.	Valid.	Train.	Test.	Valid.				
MLP 3-7-1	0.995	0.938	1.000	0.000	0.000	0.000	BFGS 3	SOS	Exponential	Tanh

*Performance term represent the coefficients of determination, while error terms indicate a lack of data for the ANN model. ANN cycles: Train. – training, Test. – testing, Valid. – validation, algor. –algorythm, funct. – function, activat. – activation.

Table S4. Elements of matrix W_1 and vector B_1 (presented in the bias column)

	1	2	3	4	5	6
Pressure	0.224	0.598	0.220	0.509	0.504	-0.096
Temperature	-0.074	-0.067	-0.138	-0.183	-0.229	0.007
CO_2 flow	0.196	0.588	0.173	0.433	0.395	-0.109
Bias	0.053	-0.095	0.015	-0.067	0.005	-0.017

Table S5. Elements of matrix W_2 and vector B_2 (presented in the bias column)

	1	2	3	4	5	6	Bias
Y	-0.206	0.142	-0.289	0.596	0.362	-0.281	-0.099

Table S6. The "goodness of fit" tests for the developed ANN model

	χ^2	RMSE	MBE	MPE	SSE	AARD	r^2
Slope (*Y*)	0.000	0.005	-0.004	27.764	$2.54 \cdot 10^{-4}$	0.049	0.974

Nomenclature

Models I-V

a - adjustable parameter

b - correction factor

f - extracted solute fraction

G - parameter related to particle size and fragmentation

k - rate constant (min^{-1}) (models I and IV)

k - rate constant (min) (model III)

K_m - mass related coefficient

$\dot{q}$ - specific CO_2 flow rate (kg CO_2/kg plant h)

Y_∞ - total yield in infinite time of extraction process (%)

t - extraction time (min)

t_1 - time constant extraction rate (min)

t_i - time of internal mass transfer (min)

Model VI

a_0 - specific interfacial area

F - correction factor for step B

H - equilibrium constant between the solid and the SCF

h - axial coordinate

k - parameter of extended Lack's model

k_f - solvent-phase mass transfer coefficient (min^{-1})

k_s - solid-phase mass transfer coefficient (min^{-1})

r - solid-phase concentration

t - time (min)

U - superficial velocity of solvent

x - concentration related to solute-free solid phase

x_0 - initial concentration of the easily accessible solute

x_k - concentration of the easily accessible solute

y - solvent-phase concentration related solute - free solvent

y_r - solubility

Y - normalized concentration

z - dimensionless coordinate

Z - parameter of fast extraction period

Greek letters

ε - void fraction

ρ - density of solvent

ρ_s - density of solid phase

τ - dimensionless time

Subscripts

k - easily accessible solute

m - start of the extraction from the inside of particles

n - end of the extraction of easily accessible solute

w - coordinate of the boundary between fast and slow extraction

Models VII and VIII

a_o - specific area per unit volume of extraction bed

c_u - asymptotic extraction yield at infinite time

D - reactor diameter

d_a - cherry seed apparent density

d_r - cherry seed real density

d_p - particle diameter,

G - initial fraction of solute in open cells

$kfa0$ - product kf · a0

$ksas$ - product ks · as

L - reactor length

m - number of experimental points,

m_{in} - cherry seed mass [g]

n - period corresponding to the end of the first extraction period

N_g - total dried mass (oil + insoluble solid)

N_{mg} - mass of insoluble solid,

q_m - q at the end of the first extraction period

q_n - q at the end of the second extraction period

r - grinding efficiency

t_i - characteristic time of the second extraction period

t' - time at the end of the first extraction period

x_u - concentration of oil in the untreated solid (oil/insoluble solid)

β_m - coefficient

γ - CO_2 to solid ratio in the bed

τ_e - external material transport resistance

τ_i - internal material transport resistance
